# Congenital intrahepatic portosystemic shunt diagnosed during intrauterine life

**DOI:** 10.1016/j.rppede.2016.03.016

**Published:** 2016

**Authors:** Camila Vieira Bellettini, Rafaela Wagner, Aleocídio Sette Balzanelo, André Luis de Souza Andretta, Arthur Nascimento de Moura, Catia Carolina Fabris, Eduardo Maranhão Gubert

**Affiliations:** a Hospital Pequeno Príncipe, Curitiba, PR, Brazil; b Pontifícia Universidade Católica do Paraná, Curitiba, PR, Brazil

**Keywords:** Prenatal diagnosis, Congenital abnormalities, CT scan, Doppler ultrasound

## Abstract

**Objective:**

To report a patient with prenatal diagnosis of portosystemic shunt; a rare condition in humans.

**Case description:**

17-Day-old female infant admitted for investigation of suspected diagnosis of portosystemic shunt, presumed in obstetric ultrasound. The hypothesis was confirmed after abdominal angiography and liver Doppler. Other tests such as echocardiography and electroencephalogram were performed to investigate possible co-morbidities or associated complications, and were normal. We chose conservative shunt treatment, as there were no disease-related complications and this was intrahepatic shunt, which could close spontaneously by the age of 2 years.

**Comments:**

Portosystemic shunt can lead to various complications such as hepatic encephalopathy, hypergalactosemia, liver tumors, and hepatopulmonary syndrome. Most diagnoses are done after one month of age, after such complications occur. The prenatal diagnosis of this patient provided greater security for the clinical picture management, as well as regular monitoring, which allows the anticipation of possible complications and perform interventional procedures when needed.

## Resumo

**Objetivo:**

Descrever a história clínica de paciente com diagnóstico pré-natal de *shunt* portossistêmico, condição rara na espécie humana.

**Descrição do caso:**

Recém-nascido do sexo feminino internada aos 17 dias para investigação de suspeita diagnóstica de *shunt* portossistêmico, aventada na ecografia obstétrica. A hipótese foi confirmada após angiotomografia do abdome e ecodoppler hepático. Outros exames, como ecocardiograma e eletroencefalograma, foram feitos para investigação de possíveis comorbidades ou complicações associadas e tiveram resultados normais. Optou-se por tratamento conservador do *shunt*, já que não havia quaisquer complicações relacionadas à doença e tratava-se de *shunt* intra-hepático, que pode fechar espontaneamente até os dois anos de idade.

**Comentários:**

O *shunt* portossistêmico pode levar a diversas complicações, como encefalopatia hepática, hipergalactosemia, tumores hepáticos e síndrome hepatopulmonar. A maioria dos diagnósticos é feita a partir de um mês de vida, após tais complicações ocorrerem. O diagnóstico pré-natal dessa paciente possibilitou maior segurança para o manejo do quadro, bem como um acompanhamento periódico que permite antecipar possíveis complicações e adotar conduta intervencionista, se necessário.

## Introduction

Portosystemic shunt is a rare condition, which was first described in 1793 by John Abernethy. It consists of congenital vascular anomaly in which the blood from the portal vein drains directly into a systemic vein, deviating from the liver circulation.^1^^,^ 2

In humans, the incidence of portosystemic shunt is estimated at one per 30,000 births and is associated with other conditions, such as gastrointestinal, genitourinary, cardiovascular, and bone malformations.^1^^-^ 3

## Case description

Newborn baby girl transferred to the service with 17 days of life for investigation of possible presence of portosystemic shunt. The hypothetical diagnosis was suggested during a routine obstetric ultrasound in the third trimester of gestation that, besides the suspected shunt, showed intrauterine growth restriction (IUGR), microcephaly, shortening of the long bones, and increased placental size. Mother had no prior or during pregnancy comorbidities.

The baby was born at 37 weeks of gestational age, via cesarean section, with birth weight 1900g, small for the gestational age,^4^ and Apgar score 6 and 9 at first and 5th minutes, respectively. At birth, she was transferred to the neonatal intensive care unit (NICU), where she remained for 11 days to gain weight and get treatment for other complications, such as respiratory distress and neonatal jaundice requiring phototherapy. After stabilization, the patient was transferred to the Hospital Pequeno Principe, in order to investigate the suspected diagnosis of portosystemic shunt.

**Figure 1:**
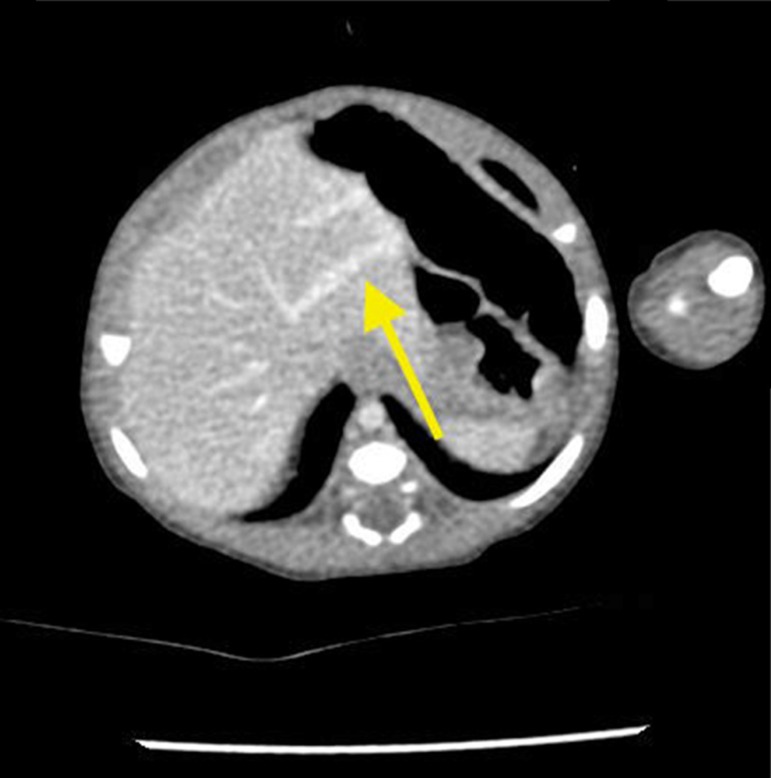
Abdominal CT angiography - venous phase. The arrow points to dilation of the intrahepatic portal vein, with portosystemic shunt.

**Figure 2:**
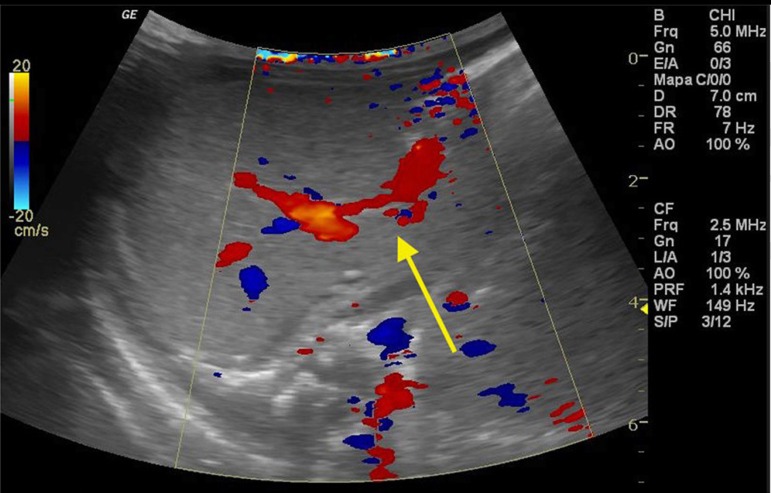
Abdominal ultrasound with Doppler. The arrow indicates the portosystemic shunt.

The newborn was admitted asymptomatic, in good general condition, ruddy, hydrated, and vital signs within normal values. Clinical examination showed no evidence of microcephaly or shortening of the long bones observed in obstetric ultrasound. The investigation continued with: (1) abdominal ultrasound, which showed no change, with insufficient assessment of portal vein; (2) computerized tomography (CT) angiography, which indicated liver with normal dimensions, contours, and density, with prominence of the left portal vein branch, up to the periphery of the left lateral segment, where it was observed vascular dilation and prominence of the adjacent left hepatic vein, with resemblance of intrahepatic portosystemic shunt on the left lateral segment of the liver (Fig. 1); (3) Abdominal ultrasound with Doppler, which showed prominences of the portal vein right and left branches, with anomalous path of right portal vein, with posterior-superior direction, communication signs between the portal vein and the middle hepatic vein, the site with increased peak systolic velocity and oscillating flow, suggestive of portosystemic shunt (Fig. 2). Because of the association of the shunt with other diseases and complications other tests were performed: normal electroencephalogram, normal echocardiogram, laboratory tests (Table 1).

**Table 1:** Laboratory tests.

Tests performed	Value found	Reference value
Albumin	3g/dL	2.8-4.4g/dL
Ammonia^a^	55µmoL/L	9-33µmoL/L
Direct bilirubin	0.48mg/dL	0.0-0.6mg/dL
Indirect bilirubin^a^	2.62mg/dL	0.2-1.0mg/dL
Total bilirubin^a^	3.1mg/dL	0.6-1.4mg/dL
Ionized calcium	1.41mmoL/L	1.15-1.32mmoL/L
Serum creatinine	0.4mg/dL	0.1-0.5mg/dL
Alkaline phosphatase^a^	385U/L	145-320U/L
Phosphorus	6.1mg/dL	3.9-6.5mg/dL
Gamma-glutamyl transferase^a^	177U/L	Even 115U/L
Hemoglobin	12.8g/dL	9.0-18.0g/dL
White blood cells	12,130µL	5000-15,000uL
Magnesium	2mg/dL	1.7-2.3mg/dL
Platelets	320,000µL	150,000-450,000µL
Potassium	5.5mmoL/L	4.1-5.3mmoL/L
C-reactive protein	6.3mg/dL	<10mg/L
Sodium	137.2mmoL/L	136-145mmoL/L
Prothrombin activity^a^	67.9%	70-100%
APTT	39.9seg (1.25)	0.8-1.25
AST	48U/L	20-60U/L
ALT	34U/L	6.0-45U/L
Urea	8mg/dL	5-40mg/dL

APTT, activated partial thromboplastin time; AST, aspartate aminotransferase; ALT, alanine aminotransferase.

a Abnormal tests.

After diagnosis, conservative treatment was chosen due to the optimal clinical outcome, absence of any disease-related complications, and type of shunt, which could close spontaneously. The child remains in periodic monitoring with clinical and laboratory assessments.

## Discussion

Portosystemic shunt is classified into intrahepatic and extrahepatic. Extrahepatic shunt directly connects the portal vein trunk (or one of its branches) with the vena cava (or one of its branches). In intrahepatic shunt occurs connection between the portal vein (or one of its branches) with the hepatic vein or inferior vena cava.^1^^,^ 3 ^,^ 5 ^,^ 6 Thus, the intestinal blood passes straight into the systemic circulation without passing through the liver.^7^

Possible factors that influence the development of this congenital malformation are: (1) genetic component; (2) complex process of malformations, with the shunt associated with heart, kidney, bone, and other malformations; (3) liver hemangioma, which can connect the vessels of portal system with hepatic vessels; and 4) absence of the ductus venosus during fetal life, which leads to the genesis of anomalous vessels to fulfill its function.^8^

Most portosystemic shunt diagnosis occurs after one month of age due to complications or even, accidentally, during the investigation of associated diseases, such as heart disease.^2^^,^ 8 In about 10% of cases, the diagnosis is made in the prenatal period, as occurred in the patient presented here.^8^ Prenatal identification of shunt has become more frequent with the technical improvement of diagnostic imaging; however, this method has limitations.^9^^,^ 10 Postnatal ultrasound is important and useful in confirming changes detected in prenatal testing.^9^

Because part of the mesenteric circulation is deviated from its passage through the liver, the metabolism of galactose, ammonia, and other toxic compounds fail to occur normally, leading to increased serum concentrations of these substances.^2^ The prevailing shunt symptoms are associated with hepatic encephalopathy resulting from this toxicity.^6^^-^ 8 ^,^ 11 Drowsiness, confusion, behavioral changes, irritability, disorientation, school difficulties, inattention and hyperactivity are the manifestations. Mental retardation and seizures may also be identified.^8^^,^ 10 Hepatic encephalopathy is more common in older patients; it is identified in 15% of cases of portosystemic shunts in children.^1^

Other serious shunt manifestations are hypergalactosemia, neonatal cholestasis, liver tumors, pulmonary arterial hypertension, hepatopulmonary syndrome.^6^^-^ 8 ^,^ 11 It may also be associated with: membranoproliferative glomerulonephritis, hyperinsulinemia with hyper- and hypoglycemia, hypothyroxinemia, hyperandrogenism, pancreatitis, and heart failure.^8^^,^ 12 There are reports in the literature suggesting that IUGR, as seen in our patient, may also be shunt-related.^8^^,^ 9 ^,^ 13 The explanation for IUGR is the reduction of liver perfusion caused by the shunt. Adequate hepatic infusion appears to induce cell proliferation with consequent increase of insulin-like growth factor-1 (IGF-1) from mRNA expression in the liver, as well as increased peripheral cell proliferation. Given the above, the literature suggests that in cases of IUGR without obvious etiology, such as placental insufficiency or chromosomal abnormalities, the possible diagnosis of portosystemic shunt should be considered and pre- and postnatal ultrasound performed.^13^

Eventually, there is liver atrophy due to decreased hepatotrophic substances entering the liver through the portal circulation.^1^^,^ 2 Portal hypertension features, such as ascites, varices, and splenomegaly, are rare in congenital portosystemic shunt. When these signs are present, other causes of spontaneous shunt should be considered.^2^ Laboratory tests may show increased levels of transaminases, gamma-glutamyl transferase, prothrombin activity, ammonia, and bilirubin. Serum albumin may be decreased.^8^ In the clinical case described here, the patient presented with minimal increased ammonia, alkaline phosphatase, gamma-glutamyl transferase and decreased prothrombin activity without any clinical consequences. These changes were not considered serious shunt complications.

The imaging test of choice for diagnosis is Doppler ultrasound of the portal system.^1^^,^ 5 ^,^ 8 CT scan is the next imaging test to be performed to better detail the region anatomy, contributing to the best therapeutic decision.^8^

Due to its severe potential complications, surgical or radiological intervention is indicated to correct the portosystemic shunt. Reports in the literature show the benefit of this repair even in asymptomatic shunts, with the role of preventing further significant repercussions.^6^^,^ 8 Moreover, the older the child and the larger the diameter of the veins involved in shunt, the harder it becomes to close it, emphasizing the importance of early intervention.^3^ On the other hand, in the specific situation of asymptomatic intrahepatic shunt in children less than 2 years old, watchful waiting is indicated, as most of them closes spontaneously before 24 months of age. However, if it does not close until the age of 2 years, intervention should be considered.^6^^,^ 8 ^-^ 10 ^,^ 13 Based on this knowledge, we opted for watchful waiting until our patient completed 2 years of age or until the appearance of signs of complications.

In symptomatic cases requiring therapeutic intervention, there are reports that, after the shunt resolution, laboratory abnormalities, neurological manifestations, and liver tumors regress completely or at least improve significantly.^8^^,^ 10 Pulmonary hypertension can be resolved, provided that the shunt is corrected before the development of irreversible vascular lesions.^8^ There is also improvement of cognitive function and recovery of weight and height in the presence of these changes.^10^

Portosystemic shunts are rare diseases with potentially serious complications. If prenatal diagnosis has not been made, it should be considered in the postnatal period in the presence of encephalopathy, pulmonary hypertension, hepatopulmonary syndrome, and neonatal cholestasis, particularly with laboratory abnormalities, such as hyperammonemia, hypergalactosemia, direct hyperbilirubinemia, and increased transaminases. In our experience, we emphasize the importance of prenatal diagnosis, which allowed greater security for the clinical picture management, as well as regular monitoring, which allows the anticipation of possible complications and perform interventional procedures when needed.
